# Experimental Evolution in *Tetrahymena*

**DOI:** 10.3390/microorganisms10020414

**Published:** 2022-02-11

**Authors:** Karissa Plum, Jason Tarkington, Rebecca A. Zufall

**Affiliations:** 1 Department of Biology and Biochemistry, University of Houston, Houston, TX 77204, USA; kplum1998@hotmail.com; 2 Department of Genetics, Stanford University, Stanford, CA 94305, USA; j.a.tarkington@gmail.com

**Keywords:** ciliates, mutation, adaptation, eco-evolutionary dynamics

## Abstract

Experimental evolution has provided novel insight into a wide array of biological processes. Species in the genus *Tetrahymena* are proving to be a highly useful system for studying a range of questions using experimental evolution. Their unusual genomic architecture, diversity of life history traits, importance as both predator and prey, and amenability to laboratory culture allow them to be studied in a variety of contexts. In this paper, we review what we are learning from experimental evolution with *Tetrahymena* about mutation, adaptation, and eco-evolutionary dynamics. We predict that future experimental evolution studies using *Tetrahyemena* will continue to shed new light on these processes.

## 1. Introduction

Experimental evolution is a powerful research approach that involves evolving populations of organisms under controlled conditions in order to test hypotheses about various evolutionary processes, such as mutation, adaptation, diversification, and coevolution [1]. Depending on the organisms used, populations may be started from isogenic lines, where all evolution is dependent on mutation to generate variation. Alternatively, initial populations may be heterogenous, with subsequent evolution resulting from the sorting of standing genetic variation. In either case, replicate populations are evolved under controlled conditions for many generations and various phenotypic, genetic, or other parameters are measured over the course of evolution. Frequently, various fitness parameters are measured in evolved populations and compared to the ancestral, or starting, populations. In more recent experiments, the ancestral and evolved populations are also often sequenced in order to determine the genomic mutations that occurred during evolution.

*Tetrahymena* are fresh-water microbial eukaryotes, belonging to the diverse group of mostly single-celled organisms known as ciliates. Similar to all ciliates, *Tetrahymena* are notable for their unusual genomic structure, where somatic and germline functions are separated into two distinct types of nuclei within a single cell. The somatic macronucleus (MAC) is the site of all transcription during vegetative growth and reproduction, but it is degraded following sexual reproduction. The germline micronucleus (MIC) is responsible for transmitting genetic information during sexual conjugation [2]. Following conjugation, the new macronucleus develops by undergoing substantial genome reorganization, including chromosomal fragmentation, the elimination of DNA, and increase in chromosome copy number [3]. This genome structure results in an unusual form of asexual division called amitosis, where chromosomes are distributed apparently randomly to offspring [4]. This genome structure has important evolutionary consequences, some of which we will highlight in this paper. *Tetrahymena* are facultatively sexual, spending most of their time reproducing asexually and only occasionally undergoing sexual conjugation. Sex is induced, at least under laboratory conditions, by starvation or other stresses, and can only occur about every 100 generations due to an immaturity period that follows conjugation [5].

*Tetrahymena* make an excellent model for experimental evolution for many reasons. First, due to their rapid generation time, it is possible to evolve *Tetrahymena* populations for many generations in a relatively short time. Short generation time is one reason that bacteria and yeast are often used in experimental evolution. *Tetrahymena* provides an interesting point of comparison to these species due to its complex genomic structure and life cycle in comparison to many of the bacteria and yeast that have been used for experimental evolution. The ability to grow *Tetrahymena* either axenically or by feeding with bacteria is also a benefit for experimental evolution [6]. Axenic cultures allow the researcher to precisely control the experimental conditions, making comparisons across replicates and/or across culture conditions more powerful. Because there is only a single species to deal with, axenic cultures also simplify genomic sequencing and analysis. However, because *Tetrahymena* are also important players in the aquatic food web as bactiverous predators and prey to larger microbes as well as fish and other animals, they can also be used to study the evolution of species interactions. Due to the fact that *Tetrahymena* has been an important model system in cell, molecular, genetic, and genomic research, there are ample resources available to facilitate the design and interpretation of experimental evolution in *Tetrahymena*. For example, having genomic sequences for the micronuclear and macronuclear genomes allows easier analysis of resequencing data [7,8,9]. Finally, the complex genomics and life cycle of *Tetrahymena* allow researchers to address questions that are difficult to address in other systems, such as the consequences of sexual versus asexual reproduction or how evolution in the germline compares to evolution in the soma.

*Tetrahymena* have been used in experimental evolution to study a variety of processes, including mutation rate, adaptation, and eco-evolutionary dynamics (Table 1). In this review, we examine how experimental evolution in *Tetrahymena* has contributed to our understanding of these evolutionary processes and we look to the future for how this system can be further used to increase our understanding of evolution. While this is not a comprehensive review of experimental evolution in *Tetrahymena*, we do hope that it conveys the utility of *Tetrahymena* as a study system for experimental evolution and provides insight into some of the fundamental questions in evolutionary biology that have been, and can be, elucidated using *Tetrahymena*.

## 2. Mutation Accumulation

Mutation is the ultimate source of all genetic variation, thus knowledge about the rate and spectrum of mutations is crucial to our understanding of evolution, disease, and biodiversity, among other things. Mutations are rare and most are deleterious, and therefore eliminated by natural selection, making it difficult to study the rate and spectrum of spontaneous mutations. To navigate this problem, an approach called mutation accumulation (MA) has been applied in many systems to study the mutational process. In an MA experiment, populations are grown for many generations to provide sufficient opportunities for mutational events. In addition, population sizes are kept as small as possible, e.g., by frequent single-cell bottlenecking. The small population size prevents natural selection from readily removing deleterious mutations and thus most mutations, except the most deleterious, have an equal chance of becoming fixed in the population due to genetic drift [10].

MA experiments have been performed in a wide variety of organisms. The usual result in these studies is that fitness declines in the MA lines due to the accumulation of deleterious mutations, confirming the prediction that most mutations that affect fitness are deleterious. By comparing the rate of decline in fitness and the variance in fitness across replicate MA lines, it is possible to estimate the rate of deleterious mutations, the average fitness effect of each mutation, and the distribution of fitness effects of new mutations [11,12,13].

By sequencing the genomes of the resulting MA lines, it is further possible to determine the rate at which various types of mutations occur, e.g., base substitutions, indels, and structural variants. In addition, mutations can be mapped to coding or noncoding regions and the genome can be assessed for the presence of mutational hot spots.

One interesting finding that has come out of a comparison of these mutation rate estimates across organisms is that mutation rate scales negatively with effective population size, consistent with the hypothesis that natural selection acts to reduce the mutation rate [14].

### 2.1. MA Experiments in Tetrahymena

The first MA experiments to be performed in a ciliate were by Brito et al. [15] on *Tetrahymena thermophila* and *T. pyriformis*. In this experiment, they found that *T. thermophila* populations quickly become extinct due to a high mutational load in the MAC resulting in a mutational meltdown [16]. They point out that it is unlikely that such rapid extinction would be due to random mutations, such as base-substitution mutations or indels. Rather, they propose that it is the gain and loss of chromosomes that occurs during amitotic division [4] that is more likely the cause of the fitness changes and extinction events. Two important lines of evidence support this conclusion. First, nucleotide-based mutations are expected, on average, to decrease population fitness; however, *Tetrahymena* populations experienced several increases in fitness that are more readily explained by the expected high rate of back mutation in copy number due to random copy number changes during amitosis. Second, Brito et al. [13] develop a model and run simulations that demonstrate that changes in chromosome copy number are sufficient to explain the observed changes in fitness and rates of extinction. In contrast, *T. pyriformis* did not become extinct during mutation accumulation, indicating a higher robustness to mutations, possibly due to the higher chromosome copy number in this species, which they confirmed by qPCR [15]. The difference between these species under MA is particularly interesting given that the species *T. pyriformis* is amicronucleate (and thus asexual), such as many other species of *Tetrahymena*, and therefore cannot “reset” its macronuclear genome by sexual conjugation [17].

Another MA experiment in *T. thermophila* took advantage of the separation of germline and somatic functions into the MIC and MAC, respectively, to obtain an unbiased estimate of mutational parameters in the MIC [18]. Because the MIC is transcriptionally silent during asexual reproduction, mutations can accumulate in the MIC without being exposed to selection, allowing for the study of mutations regardless of mutational effect, i.e., even the most deleterious mutations, which are eliminated in most microbial MA experiments, can be maintained in the MIC. In these MA experiments, cells were evolved for many generations with frequent bottlenecks. At the end of the evolution experiment, in order to study mutations in the MIC, cells were subjected to a genomic exclusion cross, allowing mutations that had accumulated in the MIC to be expressed in the MAC [6]. This study found that *T. thermophila* MIC and MAC have similar rates of mutations that affect fitness and the rate and selection coefficients of these mutations does not differ substantially from other studied eukaryotes [18].

Surprisingly, when the genomes of the MA lines from Long et al. [18] were sequenced, the base-substitution mutation rate was found to be exceptionally low [19]. This finding was consistent with the low mutation rate found in another ciliate, *Paramecium tetraurelia* [20]. It is possible that such a low mutation rate has evolved in ciliates due to strong selection on mutation rate following sexual reproduction, which results in the expression of mutations previously hidden in the MIC.

While not strictly an MA experiment, Wang et al. [21] used experimental evolution with large population sizes (see adaptation experiments below) to characterize base-substitution mutation parameters in the MAC of *T. thermophila*, and correlate them with increases in fitness. In doing so, they developed novel mutation identification and validation methodology that allows the detection of mutations at intermediate frequencies, which had not been feasible in previous experiments due to high ploidy in the MAC. This will be highly valuable for ciliate researchers pursuing this line of research in the future.

### 2.2. Future Directions in MA

These studies demonstrate the utility of *Tetrahymena* for the study of mutational parameters, consequences, and factors that drive the evolution of mutation rate. The ability to study mutations in the MIC that can remain hidden from selection throughout the course of an experiment, the availability of genetic tools such as genomic exclusion, and the diversity in genome structure and content and natural history traits across species of *Tetrahymena* makes this an excellent system in which to study mutation. Many questions remain unanswered, however. For example, the MA lines from Long et al. [19] experienced fitness declines following genomic exclusion that cannot be accounted for by MIC base-substitutions alone. Thus, it will be interesting to examine other genetic or epigenetic factors that may drive this fitness decline, such as large indels or copy number variants, or changes that occur during genome rearrangement. It is also possible that the technique used to study mutation rate in the MIC, i.e., sequencing after genomic exclusion, results in an undercounting of mutations due to RNA-based error correcting mechanisms during the development of a new MAC [22,23]. Finally, MA in *Tetrahymena* allows for the direct comparison of germline and somatic mutation parameters within a single cell. Future MA experiments in *Tetrahymena* will help to elucidate these and other questions regarding mutational processes.

## 3. Adaptation

While mutation is the ultimate source of all genetic variation, only natural selection can result in adaptation. Thus, in contrast to MA, adaptation experiments use large population sizes to maximize the effects of natural selection. Adaptive evolution experiments can therefore be used to study evolutionary dynamics, repeatability, and long-term evolutionary outcomes. These types of studies are often performed in a model bacterial system, such as *Escherichia coli,* as is the case of the pioneering work from the Lenski lab over the last three decades [24], or the model eukaryote *Saccharomyces cerevisiae* [25]. Experiments in these systems benefit from the extensive genetic and molecular toolkits that have been developed; however, an overreliance on a small number of model systems creates the risk that the outcomes of experiments in these systems are not generalizable. *Tetrahymena* species present an exciting opportunity to demonstrate whether results from experimental evolution in bacteria and yeast are applicable in a more complex eukaryote, and provide opportunities to study evolutionary scenarios that are not available in other systems.

### 3.1. Adapation Experiments in Tetrahymena

*Tetrahymena* has been used extensively over many decades to study physiological change in response to various environmental perturbations [26,27,28]. These studies have looked at changes in cellular phenotypes that are often not heritable, arise after short periods of acclimation, and tend to return to normal upon a return to standard laboratory conditions. In this section, we focus on the smaller set of studies that found heritable changes after longer periods of laboratory evolution [29,30,31,32]. These types of experiments typically utilized a serial transfer regime to culture *Tetrahymena* while maintaining large population sizes. The culture conditions are sometimes supplemented with a stressor (such as salt, ethanol, or metal) or otherwise controlled so that cells are facing a specific selection regime. In other cases, no specific stressor is employed, and cells are simply selected for high fitness conferred by, e.g., more rapid cell division.

Adaptive evolution experiments utilizing *Tetrahymena* have sought to answer questions about how the environment, population structure, genotype, or sex impact evolutionary outcomes. Often, the evolutionary outcome of interest in such studies is evolvability or the rate at which population fitness increases, however other phenotypes, such as maximum growth rate, maximum density, cell size, or membrane composition, are sometimes tracked.

#### 3.1.1. Long-Term Evolutionary Dynamics

One particularly compelling feature of experimental evolution is the ability to evolve populations in a constant environment for thousands of generations. In order to assess whether *Tetrahymena* follows similar evolutionary dynamics to other species that have been used in evolution experiments, Tarkington and Zufall [29] performed a long-term evolution experiment with *T. thermophila*. Replicate populations of different genotypes of *T. thermophila* were evolved at 2 different temperatures for 6500 generations. The maximum population growth rate was monitored for all populations at both temperatures throughout the experiment. This allowed Tarkington and Zufall [29] to ask a variety of questions about how *Tetrahymena* evolve over the long term under different conditions. This experiment demonstrated that unlike *E. coli*, where fitness continues to increase even after many thousands of generations [33], the growth rate (a proxy for fitness) of *T. thermophila* populations appeared to plateau after ~3000 generations. However, the same study found that asymmetry of the pleiotropic response across temperature regimes was similar between *E. coli* and *T. thermophila*. In both cases, populations that evolved at a hotter temperature adapted more to a colder temperature than the populations evolved at a colder temperature adapted to a hotter temperature [29,34,35]. In addition, evolution in *T. thermophila* is more repeatable, i.e., convergent among genotypes and consistent across replicates, at a hotter temperature than at a colder one. These results demonstrate that some aspects of adaptation are shared across taxa, while others differ for as yet unknown reasons.

#### 3.1.2. Adaptation to the Environment

One of the earliest laboratory evolution experiments, reported in 1964, cultured *Tetrahymena* cells in high concentrations of NaCl for over 1500 generations [32]. In this experiment researchers found that cells adapted to the high salt environment by gradually decreasing the cellular sodium concentration, which decreased by half over the course of the experiment.

Other approaches have allowed researchers to study specific molecular and biochemical responses to adaptation. In one study, Guo and Cech [36] examined how the thermostability of *Tetrahymena’s* ribozymes changed following an in vitro selection experiment using random PCR mutagenesis, which introduced an average of 2 mutations per gene at the start and then again at every third round of selection. As expected, the selected mutants had higher thermostability than wild-type ribozymes. This type of directed evolution bypasses many of the complications of traditional experimental evolution, such as the low rate of mutation, and allows for extremely high-throughput and specific selective screens to optimize specific biochemical functions; however, it is limited in its ability to answer questions about organismal adaptation and the evolutionary dynamics of natural populations.

In one traditional evolution experiment, researchers evolved *T. thermophila* for 500 generations in the lab in either stable, slowly fluctuating, or quickly fluctuating temperatures [30]. They then examined how the different evolution regimes impacted the expression of the heatshock protein, Hsp90. They found the highest levels of expression in the quickly fluctuating regime, intermediate levels of expression in the slowly fluctuating regime, and the lowest levels of expression in the stable temperature regime, demonstrating an important effect of the timescale of environmental fluctuations on evolutionary response.

Another study examined the process of adaptation of *Tetrahymena* in the presence of various heavy metals (Cd^2+^, Cu^2+^ or Pb^2+^) [37]. In this study, the concentration of the heavy metal was gradually increased once a week until a maximum tolerated concentration (MTC) was reached, after which time cells were evolved for a further 2 years at the same concentration. The ability of the strains to grow on the heavy metal increased over this period and the researchers examined how changes in the expression of a subset of metallothionein genes were involved in this adaptation. They were able to demonstrate that changes in the expression of these genes were at least partially responsible for the adaptation by utilizing knockout and knockdown strains, which displayed similar adaptive phenotypes. Work in this system also revealed a novel aspect of adaptation in *Tetrahymena*: its genomic plasticity [38]. After examining their metal adapted strains further, researchers identified at least 3 genes that had amplified their copy number ~5 fold. These amplifications were reproducible and reversible, meaning that the copy number returned to normal after evolution in the absence of the metal. This type of evolutionary adaptation may be more common in highly polyploid nuclei, similar to that of *Tetrahymena.* Additionally, the dual nuclear architecture of *Tetrahymena* also means adaptive changes in the MAC are also reversible following sex and the resetting of the MAC.

#### 3.1.3. Benefit of Sex

A few studies have also used the experimental evolution of *Tetrahymena* to investigate the role of sex in determining the adaptive outcomes. In one study, researchers set up range expansion experiments, with or without a pH gradient, and manipulated the gene flow and sexual vs. asexual modes of reproduction. They found that sex increased adaptation during range expansion in the absence of gene flow, but decreased adaptation when migration was allowed from the population core to the expanding front [39]. This was true regardless of whether an abiotic gradient was present as the range expanded, suggesting that adaptation to the low-density regime along the front can also be swamped by gene flow and sex from the non-adapted core population.

In another study, a unique benefit of sex was identified in *Tetrahymena* arising from the dual nuclear architecture and amitotic division of the polyploid MAC [40]. In this experiment, populations were either founded from a single newly produced sexual progeny or from the unmated parents. The indirect benefits of sex, which explain its prevalence despite the costs, result from the population-level increase in genetic variation [41]; however, this experiment showed that for *T. thermophila* even a single sexually produced progeny led to faster evolution than its unmated parents. This occurs because of the heterozygosity that is introduced in the polyploid MAC following sex and the subsequent allelic or phenotypic assortment, which can produce enormous amounts of combinatorial variation in the descendants of a single sexually produced progeny, allowing for rapid adaptation [4].

Another experiment explored the population dynamics of mating types in *T. thermophila*. Using a combination of experimental evolution and population modeling, Wang et al. [42] found that if not all cells undergo conjugation during a period of mating, selection will always result in the fixation of a mating type associated with a beneficial allele via genetic hitchhiking. This result is particularly interesting, given the unusual mechanism of sex determination in *T. thermophila*, i.e., caryonidal inheritance or probabilistic sex determination [43,44], and the distribution of mating types in natural populations, i.e., that all ponds studied so far contain all seven mating types. [45].

Interestingly, many species of *Tetrahymena* are incapable of sexual reproduction and some have been asexual for millions of years [17]. The success of asexuality in this genus may be, in part, due to the fact that amitosis allows for the generation of variation among asexual progeny, unlike other taxa which require sexual reproduction to generate variable progeny [46]. Nonetheless, the precise costs and benefits of sex in this genus, and in ciliates more broadly, remain unclear.

### 3.2. Future Directions in Adapation Experiments

These experiments demonstrate the wide variety of biological phenomena that can be examined in *Tetrahymena* evolving in the lab. *Tetrahymena* allow for important comparisons across taxa in the processes that determine adaptive trajectories and outcomes, and allow for a unique insight into the effects of germ-soma differentiation and genome rearrangement. Given the abundance and importance of *Tetrahymena*, and ciliates more broadly, in aquatic habitats [47], it seems that experimental evolution will be a powerful tool to study how they will respond to our rapidly changing environment. In addition, we predict that we will gain unprecedented insight into the mechanisms underlying adaptation, as more genomic tools, such as long-read sequencing to study copy number variation, are applied to these adaptation experiments.

## 4. Eco-Evolutionary Dynamics

Eco-evolutionary dynamics describe the effects of ecological processes on evolutionary change, the effects of evolutionary change on ecological processes, and the feedback between ecological and evolutionary changes. It has long been understood that ecology can influence evolution (e.g., by driving adaption), and vice versa, however the formal study of these eco-evolutionary dynamics is relatively recent [48]. Pelletier et al. [48] identified some central questions that an eco-evolutionary approach can address, e.g., how does evolution influence demography, how do ecological conditions influence the potential for evolution, and how can evolution influence community and ecosystem traits? Answers to these, and related, questions will help provide an explanation for the extensive organismal diversity found in nature.

### 4.1. Eco-Evolutionary Dynamics Experiments in Tetrahymena

Since the effects of evolution on ecology, or ecology on evolution, may not be evident until after many generations, microbes present useful models to study these dynamics. With a fast generation time and ease of culturing in the lab, *Tetrahymena* can be used to study eco-evolutionary dynamics over many generations in a short period of time, with a large number of replicate populations. *Tetrahymena* have many additional useful features for studying ecological processes. For example, *Tetrahymena* are effective bacterial predators [47], and one widely used system in the field of eco-evolutionary dynamics is the *Tetrahymena thermophila*–*Pseudomonas fluorescens* predator–prey system. Although this model may be simple relative to the complex set of interactions that occur in natural populations, it nonetheless provides a valuable insight into complex predator–prey dynamics. *Tetrahymena* are also useful because they possess genetic diversity in dispersal patterns [49] and can grow in a variety of environmental conditions [31,32,50], allowing for the possibility of many different questions to be explored. As a result of this versatility, many different types of questions about eco-evolutionary dynamics can be addressed using *Tetrahymena*, and many of these studies have important implications for conservation efforts.

#### 4.1.1. Single-Species Experiments

Eco-evolutionary dynamics studies with a single species, i.e., *Tetrahymena* by itself, are similar to adaptation experiments described above. These experiments are useful for exploring the various types of ecological and evolutionary processes that are likely to interact. For example, by evolving *T. thermophila* under low pH and high population densities, Moerman et al. [31] demonstrated that evolutionary outcomes are affected by demography. They found that four different genotypes evolved under the same conditions all experienced a convergence of intrinsic rates of increase (*r*_0_) and intraspecific competitive ability (α), demonstrating selection on density-dependent fitness.

Other studies investigating eco-evolutionary dynamics of *Tetrahymena* in a single-species context have focused on range expansions. Fronhofer and Altermatt [51] found that the ecological process of range expansions leads to the evolution of increased dispersal, which then feeds back on ecological patterns affecting the spatial distribution of population densities. In a subsequent experiment, they demonstrated that information provided in the form of environmental gradients altered the eco-evolutionary dynamics of range expansions previously observed [52]. They emphasized consideration of eco-evolutionary dynamics and the factors that could affect those dynamics for conservation efforts.

#### 4.1.2. Multi-Species Experiments

Eco-evolutionary dynamics studies have also used two-species systems to investigate predator–prey interactions, where a species of *Tetrahymena* (usually *thermophila* or *vorax)* is used as a predator of a bacterial species, either *Pseudomonas fluorescens* or *Serratia marcescens*. To demonstrate the existence of eco-evolutionary dynamics, Friman et al. [53] show that ecological processes affect evolutionary processes and vice versa using a predator–prey community composed of two strains of *P. fluorescens* and *T. thermophila*. Their results show that rapid prey evolution can alter the structure of predator–prey communities by acting on initial genetic variation in prey, which in turn affects the evolutionary trajectory of a community.

Other studies using *Tetrahymena* as a predator of a bacterial species, investigate how various factors can alter eco-evolutionary dynamics of the system. Two studies have assessed the role of resource availability in shaping eco-evolutionary dynamics of the *T. thermophila–S. marcescens* predator–prey system. Both studies showed that resource availability altered eco-evolutionary dynamics through decreased predator defense in prey, which in turn led to changes in the population density of predator and prey [54,55]. In another study, the presence of antibiotics in a *T. thermophila*–*P. fluorescens* community was shown to slow evolution of anti-predator defenses and antibiotic resistance, which in turn altered the ecological dynamics [56]. These studies thus demonstrate that external stressors have substantial effects on the eco-evolutionary dynamics of a system.

The presence of multiple stressors on the *T. thermophila*–*P. fluorescens* system were also investigated. Hiltunen et al. [57] simulated fluctuating environments with bottom-up stressors (resource availability affecting prey *P. fluorescens*), top-down stressors (salinity affecting predator *T. thermophila*), or combinations (synchronous and asynchronous) of these stressors. Their results demonstrated that the rate of evolution of defense was significantly lower in fluctuating compared with stable environments and that the defense evolved to lower levels when two environmental stressors recurrently changed. This was explained by fluctuations in population sizes of both the prey and predator.

Another study tested the effect of co-evolution on the eco-evolutionary dynamics of the *T. thermophila*–*P. fluorescens* system. Hiltunen and Becks [58] found that predators that were co-evolved with the prey showed important differences in evolutionary and ecological parameters, e.g., a faster evolution of defense traits and higher carrying capacity. Further, they found that the changes in predator traits in the co-evolved populations shifted the dynamics from evolution driving ecology, to ecology driving evolution.

While two-species communities are a useful starting point for studying community dynamics, often two species are not enough to understand an entire community. By building off the protist predator–bacteria prey system, researchers have been able to show that community dynamics become more complicated as more species are added. One study exposed *P. fluorescence* to multiple species of protist predators, including two species of *Tetrahymena*, in different combinations. They found that predator community composition affects both ecological and evolutionary processes and can determine when rapid evolution will change the ecological properties of microbial communities [59]. Another study used a different multiple predator–prey system that included *S. marcescens* (prey)*, T. thermophila* and *Acanthamoeba castellanii* (predators), as well as a bacteriophage. They also repeated the experiment with *P. fluorescence* instead of *S. marcescens* to verify the results. They found that bacteria that had evolved with both enemies were overall less susceptible to infection by both ancestral and co-evolved phages, suggesting an overlap between defense mechanisms for predators and phages [60].

Researchers have also used multi-species systems to study additional phenomena that had previously been studied in the two-species system. Hiltunen et al. [61] assessed the role of resource availability on eco-evolutionary dynamics of a community by exposing communities composed of three bacterial prey species and a single predator species, *T. thermophila,* to fluctuations in resource availability. They found that both resource fluctuations and predation increased community evenness, and that the two interacted to promote evenness as well. Another study investigated the effects of trait variation in both predator (co-evolved vs. naïve) and prey (multiple colonies) on the eco-evolutionary dynamics of a community [62]. They found that initial trait variation in both predator and prey enhanced coexistence of the community. Both of these studies highlight the importance of eco-evolutionary dynamics in conservation efforts.

### 4.2. Future Directions in Eco-Evolutionary Dynamics Experiments

While many studies have investigated eco-evolutionary dynamics, there are still many unexplored avenues to be addressed in future studies. For example, many important environmental factors have not been examined. Given that global temperatures continue to rise, studies of *Tetrahymena* predator–prey dynamics will be informative in understanding how communities will respond under this stress. Another important factor is the effect of common environmental toxins on these systems. It will also be informative to explore how multiple stressors in tandem effect different communities. Lastly, it is important that future studies include more than two or three species. While adding more species to a study system can present complications to interpreting results, systems with fewer species may not adequately represent what is happening in nature. As with the adaptation experiments described above, we also predict here that genomic tools, such as RNAseq, applied to eco-evolutionary dynamics experiments will elucidate the underlying genetic mechanisms contributing to the observed evolutionary and ecological outcomes.

## 5. Summary

*Tetrahymena* has long been an important study system in molecular and cellular biology and genetics (reviewed in [63]). However, despite some early adaptation experiments in this group, only recently has it been recognized as an important system for experimental evolution. Studies employing *Tetrahymena* have since illuminated both phenomena unique to *Tetrahymena* (or ciliates), e.g., a low mutation rate, and broader evolutionary patterns, e.g., the benefits of sex. We predict that the unusual biological features of *Tetrahymena*, coupled with the ease of manipulation under laboratory conditions, will allow for continued discovery using various experimental evolution approaches with *Tetrahymena*.

## Figures and Tables

**Table 1:** Types of experimental evolution research that have employed *Tetrahymena*.

Type of Experiment	Population Size	Predominant Evolutionary Force	Traits Measured
Mutation accumulation	Small	Mutation, genetic drift	Fitness, genetic mutations
Adaptation	Large	Selection	Fitness, population dynamics, other phenotypes
Eco-evolutionary dynamics	Medium to large	Coevolution, selection	Ecological dynamics, adaptive traits

## References

[1] Kawecki T.J., Lenski R.E., Ebert D., Hollis B., Olivieri I., Whitlock M.C. (2012). Experimental evolution. Trends Ecol. Evol..

[2] Prescott D.M. (1994). The DNA of ciliated protozoa. Microbiol. Rev..

[3] McGrath C.L., Zufall R.A., Katz L.A. (2006). Genome evolution in ciliates. Genomics and Evolution of Microbial Eukaryotes.

[4] Doerder F.P., Deak J.C., Lief J.H. (1992). Rate of phenotypic assortment in *Tetrahymena thermophila*. Dev. Genet..

[5] Lynn D.H., Doerder F.P., Collins K. (2012). Chapter 2—The Life and Times of *Tetrahymena*. Methods in Cell Biology.

[6] Cassidy-Hanley D.M., Collins K. (2012). Chapter 8—*Tetrahymena* in the Laboratory: Strain Resources, Methods for Culture, Maintenance, and Storage. Methods in Cell Biology.

[7] Hamilton E.P., Kapusta A., Huvos P.E., Bidwell S.L., Zafar N., Tang H., Hadjithomas M., Krishnakumar V., Badger J.H., Caler E.V. (2016). Structure of the germline genome of *Tetrahymena thermophila* and relationship to the massively rearranged somatic genome. eLife.

[8] Eisen J.A., Coyne R.S., Wu M., Wu D., Thiagarajan M., Wortman J.R., Badger J.H., Ren Q., Amedeo P., Jones K.M. (2006). Macronuclear genome sequence of the ciliate *Tetrahymena thermophila*, a model eukaryote. PLoS Biol..

[9] Sheng Y., Duan L., Cheng T., Qiao Y., Stover N.A., Gao S. (2020). The completed macronuclear genome of a model ciliate *Tetrahymena thermophila* and its application in genome scrambling and copy number analyses. Sci. China Life Sci..

[10] Halligan D.L., Keightley P.D. (2009). Spontaneous Mutation Accumulation Studies in Evolutionary Genetics. Annu. Rev. Ecol. Evol. Syst..

[11] Bateman A.J. (1959). The Viability of Near-normal Irradiated Chromosomes. Int. J. Radiat. Biol. Relat. Stud. Phys. Chem. Med..

[12] Mukai T. (1964). The Genetic Structure of Natural Populations of *Drosophila Melanogaster*. I. Spontaneous Mutation Rate of Polygenes Controlling Viability. Genetics.

[13] Keightley P.D. (1994). The distribution of mutation effects on viability in *Drosophila melanogaster*. Genetics.

[14] Sung W., Ackerman M.S., Miller S.F., Doak T.G., Lynch M. (2012). Drift-barrier hypothesis and mutation-rate evolution. Proc. Natl. Acad. Sci. USA.

[15] Brito P.H., Guilherme E., Soares H., Gordo I. (2010). Mutation accumulation in *Tetrahymena*. BMC Evol. Biol..

[16] Lynch M., Conery J., Burger R. (1995). Mutational Meltdowns in Sexual Populations. Evolution.

[17] Doerder F.P. (2014). Abandoning sex: Multiple origins of asexuality in the ciliate *Tetrahymena*. BMC Evol. Biol..

[18] Long H.-A., Paixão T., Azevedo R.B.R., Zufall R.A. (2013). Accumulation of spontaneous mutations in the ciliate *Tetrahymena thermophila*. Genetics.

[19] Long H., Winter D.J., Chang A.Y.-C., Sung W., Wu S.H., Balboa M., Azevedo R.B.R., Cartwright R.A., Lynch M., Zufall R.A. (2016). Low base-substitution mutation rate in the germline genome of the ciliate *Tetrahymena thermophila*. Genome Biol. Evol..

[20] Sung W., Tucker A.E., Doak T.G., Choi E., Thomas W.K., Lynch M. (2012). Extraordinary genome stability in the ciliate *Paramecium tetraurelia*. Proc. Natl. Acad. Sci. USA.

[21] Wang G., Fu L., Xiong J., Mochizuki K., Fu Y., Miao W. (2021). Identification and Characterization of Base-Substitution Mutations in the Macronuclear Genome of the Ciliate Tetrahymena thermophila. Genome Biol. Evol..

[22] Nowacki M., Vijayan V., Zhou Y., Schotanus K., Doak T.G., Landweber L.F. (2008). RNA-mediated epigenetic programming of a genome-rearrangement pathway. Nature.

[23] Fang W., Landweber L.F. (2013). RNA-mediated genome rearrangement: Hypotheses and evidence. Bioessays.

[24] Lenski R.E., Rose M.R., Simpson S.C., Tadler S.C. (1991). Long-Term Experimental Evolution in *Escherichia coli*. I. Adaptation and Divergence during 2000 Generations. Am. Nat..

[25] Levy S.F., Blundell J.R., Venkataram S., Petrov D.A., Fisher D.S., Sherlock G. (2015). Quantitative evolutionary dynamics using high-resolution lineage tracking. Nature.

[26] Hirobumi F., Martin C.E., Hisaya I., Yasuo K., Thompson G.A., Yoshinori N. (1976). Changes in membrane lipid composition during temperature adaptation by a thermotolerant strain of *Tetrahymena pyriformis*. Biochim. Biophys. Acta (BBA)-Lipids Lipid Metab..

[27] Rasmussen L., Toftlund H., Suhr-Jessen P. (1984). *Tetrahymena*. Adaptation to high iron. Exp. Cell Res..

[28] Nandini-Kishore S.G., Mattox S.M., Martin C.E., Thompson G.A. (1979). Membrane changes during growth of *Tetrahymena* in the presence of ethanol. Biochim. Biophys. Acta.

[29] Tarkington J., Zufall R.A. (2021). Temperature affects the repeatability of evolution in the microbial eukaryote *Tetrahymena thermophila*. Ecol. Evol..

[30] Ketola T., Laakso J., Kaitala V., Airaksinen S. (2004). Evolution of Hsp90 expression in *Tetrahymena thermophila* (Protozoa, Ciliata) populations exposed to thermally variable environments. Evolution.

[31] Moerman F., Arquint A., Merkli S., Wagner A., Altermatt F., Fronhofer E.A. (2020). Evolution under pH stress and high population densities leads to increased density-dependent fitness in the protist *Tetrahymena thermophila*. Evolution.

[32] Dunham P.B. (1964). The adaptation of *Tetrahymena* to a high NaCl environment. Biol. Bull..

[33] Lenski R.E., Wiser M.J., Ribeck N., Blount Z.D., Nahum J.R., Morris J.J., Zaman L., Turner C.B., Wade B.D., Maddamsetti R. (2015). Sustained fitness gains and variability in fitness trajectories in the long-term evolution experiment with *Escherichia coli*. Proc. R. Soc. B Biol. Sci..

[34] Bennett A.F., Lenski R.E. (1993). Evolutionary adaptation to temperature II. Thermal niches of experimental lines of *Escherichia coli*. Evolution.

[35] Mongold J.A., Bennett A.F., Lenski R.E. (1996). Evolutionarey adaptation to temperature. IV. Adaptation of *Escherichia coli* at a niche boundary. Evolution.

[36] Guo F., Cech T.R. (2002). Evolution of Tetrahymena ribozyme mutants with increased structural stability. Nat. Struct. Mol. Biol..

[37] de Francisco P., Martín-González A., Turkewitz A.P., Gutiérrez J.C. (2017). Extreme metal adapted, knockout and knockdown strains reveal a coordinated gene expression among different *Tetrahymena thermophila* metallothionein isoforms. PLoS ONE.

[38] de Francisco P., Martín-González A., Turkewitz A.P., Gutiérrez J.C. (2018). Genome plasticity in response to stress in *Tetrahymena thermophila*: Selective and reversible chromosome amplification and paralogous expansion of metallothionein genes. Environ. Microbiol..

[39] Moerman F., Fronhofer E.A., Wagner A., Altermatt F. (2020). Gene swamping alters evolution during range expansions in the protist *Tetrahymena thermophila*. Biol. Lett..

[40] Tarkington J., Zhang H., Azevedo R.B.R., Zufall R.A. (2021). Sex, amitosis, and evolvability in the ciliate *Tetrahymena thermophila*. bioRxiv.

[41] Burt A. (2000). Perspective: Sex, Recombination, and the Efficacy of Selection—Was Weismann Right?. Evolution.

[42] Wang G., Chen K., Zhang J., Deng S., Xiong J., He X., Fu Y., Miao W. (2020). Drivers of Mating Type Composition in *Tetrahymena thermophila*. Genome Biol. Evol..

[43] Nanney D.L. (1956). Caryonidal inheritance and nuclear differentiation. Am. Nat..

[44] Phadke S.S., Zufall R.A. (2009). Rapid diversification of mating systems in ciliates. Biol. J. Linn. Soc..

[45] Doerder F.P., Gates M.A., Eberhardt F.P., Arslanyolu M. (1995). High-Frequency of Sex and Equal Frequencies of Mating Types in Natural-Populations of the Ciliate *Tetrahymena thermophila*. Proc. Natl. Acad. Sci. USA.

[46] Zhang H., West J.A., Zufall R.A., Azevedo R.B.R. (2021). Amitosis confers benefits of sex in the absence of sex to Tetrahymena. bioRxiv.

[47] Sherr E.B., Sherr B.F. (2002). Significance of predation by protists in aquatic microbial food webs. Antonie Leeuwenhoek.

[48] Pelletier F., Garant D., Hendry A.P. (2009). Eco-evolutionary dynamics. Philos. Trans. R. Soc. B Biol. Sci..

[49] Pennekamp F., Mitchell K.A., Chaine A., Schtickzelle N. (2014). Dispersal Propensity in *Tetrahymena thermophila* Ciliates—A Reaction Norm Perspective. Evolution.

[50] Maurya R., Pandey A.K. (2020). Importance of protozoa *Tetrahymena* in toxicological studies: A review. Sci. Total Environ..

[51] Fronhofer E.A., Altermatt F. (2015). Eco-evolutionary feedbacks during experimental range expansions. Nat. Commun..

[52] Fronhofer E.A., Nitsche N., Altermatt F. (2017). Information use shapes the dynamics of range expansions into environmental gradients. Glob. Ecol. Biogeogr..

[53] Friman V.-P., Jousset A., Buckling A. (2014). Rapid prey evolution can alter the structure of predator–prey communities. J. Evol. Biol..

[54] Friman V.-P., Hiltunen T., Laakso J., Kaitala V. (2008). Availability of prey resources drives evolution of predator–prey interaction. Proc. R. Soc. B Biol. Sci..

[55] Friman V.-P., Laakso J. (2011). Pulsed-Resource Dynamics Constrain the Evolution of Predator-Prey Interactions. Am. Nat..

[56] Hiltunen T., Cairns J., Frickel J., Jalasvuori M., Laakso J., Kaitala V., Künzel S., Karakoc E., Becks L. (2018). Dual-stressor selection alters eco-evolutionary dynamics in experimental communities. Nat. Ecol. Evol..

[57] Hiltunen T., Ayan G.B., Becks L. (2015). Environmental fluctuations restrict eco-evolutionary dynamics in predator–prey system. Proc. R. Soc. B Biol. Sci..

[58] Hiltunen T., Becks L. (2014). Consumer co-evolution as an important component of the eco-evolutionary feedback. Nat. Commun..

[59] Friman V.-P., Dupont A., Bass D., Murrell D.J., Bell T. (2016). Relative importance of evolutionary dynamics depends on the composition of microbial predator–prey community. ISME J..

[60] Örmälä-Odegrip A.-M., Ojala V., Hiltunen T., Zhang J., Bamford J.K., Laakso J. (2015). Protist predation can select for bacteria with lowered susceptibility to infection by lytic phages. BMC Evol. Biol..

[61] Hiltunen T., Friman V.-P., Kaitala V., Mappes J., Laakso J. (2012). Predation and resource fluctuations drive eco-evolutionary dynamics of a bacterial community. Acta Oecologica.

[62] Scheuerl T., Cairns J., Becks L., Hiltunen T. (2019). Predator coevolution and prey trait variability determine species coexistence. Proc. R. Soc. B.

[63] Ruehle M.D., Orias E., Pearson C.G. (2016). *Tetrahymena* as a Unicellular Model Eukaryote: Genetic and Genomic Tools. Genetics.

